# Nonlinear Optical Characterization of InP@ZnS Core-Shell Colloidal Quantum Dots Using 532 nm, 10 ns Pulses

**DOI:** 10.3390/nano11061366

**Published:** 2021-05-21

**Authors:** Rashid A. Ganeev, Andrey I. Zvyagin, Ivan A. Shuklov, Maksim G. Spirin, Oleg V. Ovchinnikov, Vladimir F. Razumov

**Affiliations:** 1 Department of Physics, Voronezh State University, 394006 Voronezh, Russia; andzv92@yandex.ru (A.I.Z.); ovchinnikov_o_v@rambler.ru (O.V.O.); 2 Laboratory for Photonics of Quantum Nanostructures, Moscow Institute of Physics and Technology (National Research University), 141701 Dolgoprudny, Russia; max2004@icp.ac.ru (M.G.S.); razumovvf@list.ru (V.F.R.); 3 Institute of Astronomy, University of Latvia, 1586 Riga, Latvia; 4 Department of Physics, American University of Sharjah, Sharjah 26666, United Arab Emirates; 5 Institute of Problems of Chemical Physics, Russian Academy of Sciences, 142432 Chernogolovka, Russia

**Keywords:** core-shell colloidal quantum dots, InP@ZnS, nonlinear absorption, nonlinear refraction, saturable absorption

## Abstract

InP@ZnS core-shell colloidal quantum dots (CQDs) were synthesized and characterized using the z-scan technique. The nonlinear refraction and nonlinear absorption coefficients (*γ* = −2 × 10^−12^ cm^2^ W^−1^, *β* = 4 × 10^−8^ cm W^−1^) of these CQDs were determined using 10 ns, 532 nm pulses. The saturable absorption (*β* = −1.4 × 10^−9^ cm W^−1^, *I*_sat_ = 3.7 × 10^8^ W cm^−2^) in the 3.5 nm CQDs dominated at small intensities of the probe pulses (*I* ≤ 7 × 10^7^ W cm^−2^) followed by reverse saturable absorption at higher laser intensities. We report the optical limiting studies using these CQDs showing the suppression of propagated nanosecond radiation in the intensity range of 8 × 10^7^–2 × 10^9^ W cm^−2^. The role of nonlinear scattering is considered using off-axis z-scan scheme, which demonstrated the insignificant role of this process along the whole range of used intensities of 532 nm pulses. We discuss the thermal nature of the negative nonlinear refraction in the studied species.

## 1. Introduction

Interest in nanoparticles is related with their potential applications in laser physics, optoelectronics, medicine, etc. Among studied species are the depositions of metal nanoparticles in dielectrics [1,2,3,4], semiconductor nanoparticles synthesized during laser ablation in liquids [5], chalcogenide nanoparticles doped in thin films [6], etc. During last two decades, the small-sized nanoparticles, like quantum dots, became an important subject of studies.

Quantum dots (QDs) offer the fundamental and practical interest due to the size-related optical characteristics. Among various applications are the quantum computing, high-order harmonics generation, enhancement of photodiodes efficiency, amendments of LEDs and solar cells, microscopy, etc. The least studied are the nonlinear optical properties of QDs. There was a limited account during most of previous research of QDs for the propagation phenomena, like dispersion, two-photon absorption (TPA), saturable absorption (SA), reverse saturable absorption (RSA), high-order harmonic generation, and the Kerr effect causing the nonlinear refraction. In most cases, the non-resonance conditions were explored when the excitonic band of semiconductor-based QDs was far from the wavelengths of the probe pulses.

Of great interest for the analysis of the nonlinear optical response are the core-shell QDs, which is attributed to the modulation of different properties of core components by the shell particles [7,8]. On the one hand, the formation of core-shell QDs leads to a significant transformation of the structure of local states within the effective band gap of the crystal core. This process affects the transformation of the properties of nonlinear absorption and nonlinear refraction resulting from transitions involving real energy levels of structural impurity defects. On the other hand, core-shell QDs, with appropriate selection of the energy levels of the core and shell, can lead to effective charge separation during photoexcitation. The processes of charge phototransfer can radically change the nonlinear optical properties of core-shell QDs. For example, strong nonlinear optical properties have been demonstrated in the case of binary nanocomposites (Ag_2_S-CuS, Ag_2_S-CdS, Ag_2_S-ZnS, Ag_2_S-graphene, etc. [9,10,11]).

The object attracting attention during last time is the combination of InP and ZnS (InP@ZnS) core-shell nanocomposites. Each of components of the InP@ZnS structure possesses their own particular nonlinear optical properties. The nonlinear optical studies of InP nanowires were reported in [12]. Most of previous studies of InP@ZnS QDs were carried out using ultrafast lasers (130 fs [13] and 100 fs [14]). The application of longer probe pulses (i.e., those in the range of a few nanoseconds) can reveal new peculiarities in the low-order nonlinear optical response of these components and the compounds comprising InP and ZnS small-sized particles. It is interesting to analyze to which extent some of the nonlinear optical properties of this complex system mimic or enhance the properties of its components due to different response of the ingredients of this system once using significantly longer (nanosecond) probe pulses. The resistance to photobleaching is another attractive feature of QDs [15,16]. The saturable absorption properties can be modified in InP@ZnS core-shell QDs in the case of probing by 10-ns-class lasers.

Here we report the synthesis of 3.5 nm InP@ZnS core-shell QDs and characterize them using the z-scan technique allowing the analysis of the low-order optical nonlinearities using long pulses. We demonstrate that this core-shell structure possesses strong nonlinear absorption and thermal-lens-related nonlinear refraction at the wavelength of *λ* = 532 nm in the case of 10 ns laser pulses. We show that saturable absorption dominates in these QDs at small intensities of the probe pulses followed by reverse saturable absorption at higher laser intensities. The nonlinear optical response of InP@ZnS colloidal quantum dots (CQDs) in the case of 532 nm pulses, the optical limiting studies, the mechanisms of saturable absorption, nonlinear refraction, and nonlinear scattering of InP@ZnS CQDs are studied and discussed.

## 2. Synthesis and Characterization of InP@ZnS CQDs

Synthesis of colloidal core-shell quantum dots could be performed in various manners [17,18]. The detailed synthesis process of the InP@ZnS QDs is reported in Ref. [19]. Briefly, the procedure was as follows. The following reagents were used for the synthesis of InP@ZnS CQDs: indium chloride (99.995%, Acros, Geel, Belgium), zinc chloride (anhydrous, 98%, Sigma-Aldrich, Taufkirchen, Germany), oleylamine (OLA, 80–90%, Acros, Geel, Belgium), tris(dimethylamino)phosphine (97%, Sigma-Aldrich, Taufkirchen, Germany), 1-dodecanetiol (DDT, 98%, Sigma-Aldrich, Taufkirchen, Germany), chloroform (99.5%, Sigma-Aldrich, Taufkirchen, Germany, 0.01–0.02% of amylenes), and methanol (reagent grade, Khimmed, Moscow, Russia). InP cores were obtained using the method described in [20]. For this purpose, a mixture of InCl_3_ (0.9 mmol), ZnCl_2_ (0.9 mmol) and amine (15.2 mmol) was placed in a reaction vessel and degassed at 110 °C for 40 min. After that the temperature of the mixture was increased to 220 °C in an argon atmosphere and then the phosphorus precursor (TDMAP, 1.4 mmol) was added to the mixture. In the presence of this reagent, nuclei with an average diameter of 3 nm were formed within 7 min. To grow the ZnS shell, sulfur precursor (DDT, 10.6 mmol) was introduced into the prepared mixture.

After that the growth of the nuclei almost stopped, and their size was estimated from spectral data using the method described in [21]. Then the temperature in the reactor was reduced to 200 °C and kept for 1 h. Two LED light sources (Ocean Optics, Orlando, USA, LLS-455, *λ* = 455 nm) were installed at an angle of 90° and at a separation of 0.17 m from the reactor with a reflecting screen behind. To complete the process, the mixture was quickly cooled by feeding water into the jacket of the reaction vessel. When reaching 110 °C, 10 mL of toluene was added directly to the reactor. For subsequent use, the obtained CQDs were washed with methanol (1:1 vol.), deposited in a centrifuge (3000 min^−1^), dried, dispersed in chloroform, and filtered through membranes with a pore diameter of 450 nm.

**Figure 1:**
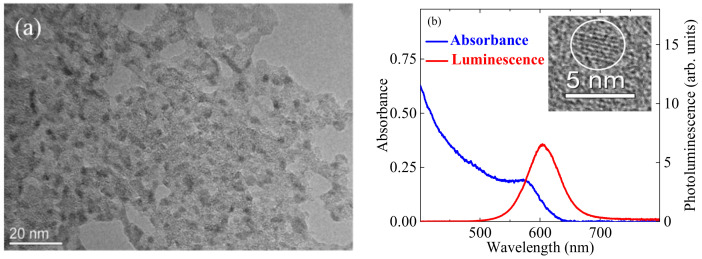
(**a**) TEM image of InP@ZnS QDs. (**b**) Absorption (blue curve) and photoluminescence (red curve) spectra of InP@ZnS CQDs. The absorption spectrum of InP@ZnS shows first excitonic peak at ~575 nm. The emission band excited by 400 nm radiation was centered at 607 nm, with full width at half-maximum of 70 nm. Inset: High resolution TEM image of QD.

Transmission electron microscopy (TEM, JEOL JEM-2100 microscope, Tokyo, Japan) and high resolution TEM of single QD with mean diameter d = 3.5 nm are shown in Figure 1a and in the inset to Figure 1b, respectively. The results of spectroscopic studies are presented in Figure 1b, which show the absorption (blue curve) and photoluminescence (red curve) spectra of InP@ZnS QDs suspension. Blue curve refers to conventionally (no activation) prepared CQDs. The absorption spectrum exhibits the presence of excitonic bands of InP cores in the range 550–600 nm centered at ~575 nm. The band gap of InP@ZnS QDs was estimated to be 1.89 eV. The luminescence spectrum shows the symmetrical band peaked at 607 nm (red curve) typical for excitonic irradiation.

The determination of the formation of core-shell structure was performed by the following procedure. TEM microphotographs showed the growth of particles during the build-up of the shell [19]. In that case the lesser change of absorption spectrum compared with the case of the growth of indium phosphide nuclei was observed [20]. At the same time, the luminescence was greatly increased, which is typical for the structures like InP-ZnS core-shell QDs [22].

Studies of the nonlinear optical properties of QDs were performed using standard z-scan technique [23]. The second harmonic (λ = 532 nm) of the Nd:YAG laser (pulse duration 10 ns, pulse repetition rate 1 Hz) was focused using a 300 mm focal length spherical lens. The beam diameter at input lens was ~10 mm (FW1/e^2^M). The intensity distribution of laser beam was close to the Gaussian shape. M^2^ value was calculated to be 1.4. The beam waist diameter was 60 μm. The 1-mm-thick fused silica cell containing CQDs was moved along the *z*-axis through the focal point using a translating stage controlled by a computer. The energies of the initial and propagated laser pulses were measured using the calibrated photodiodes. The closed-aperture (CA) and open-aperture (OA) schemes allowed determination of the nonlinear refraction indices (*γ*) and nonlinear absorption coefficients (*β*) of the samples, respectively.

The optical limiting (OL) studies were carried out by varying the energy of the pulses propagating through the CQD-contained cell installed in the focal plane. The energy was changed using the calibrated filters. We also carried out the nonlinear scattering studies of InP@ZnS CQDs.

## 3. Z-Scans of InP@ZnS CQDs

**Figure 2:**
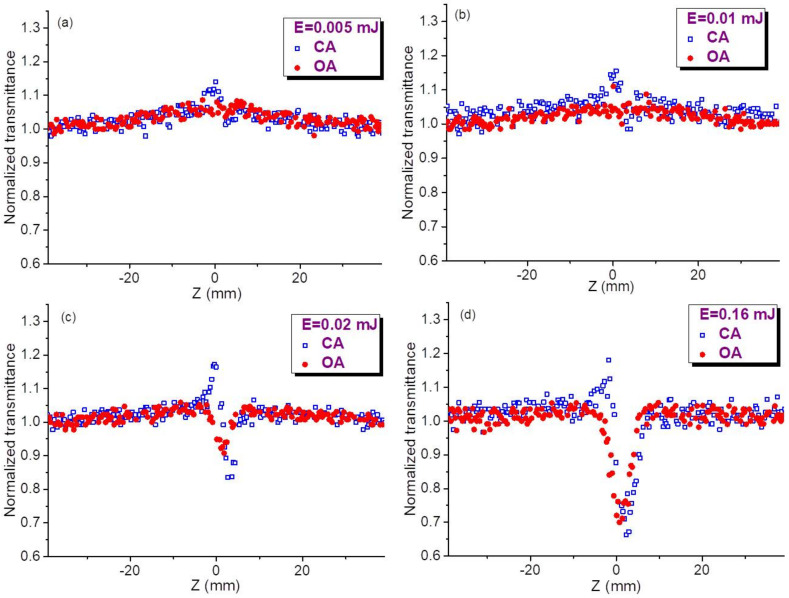
Open-aperture (OA, red filled circles) and closed-aperture (CA, blue empty squares) z-scans of InP@ZnS CQDs (*C* = 2 × 10^−6^ M) measured at different energies of 532 nm pulses. (**a**) 0.005 mJ, (**b**) 0.01 mJ, (**c**) 0.02 mJ, (**d**) 0.16 mJ.

**Figure 3:**
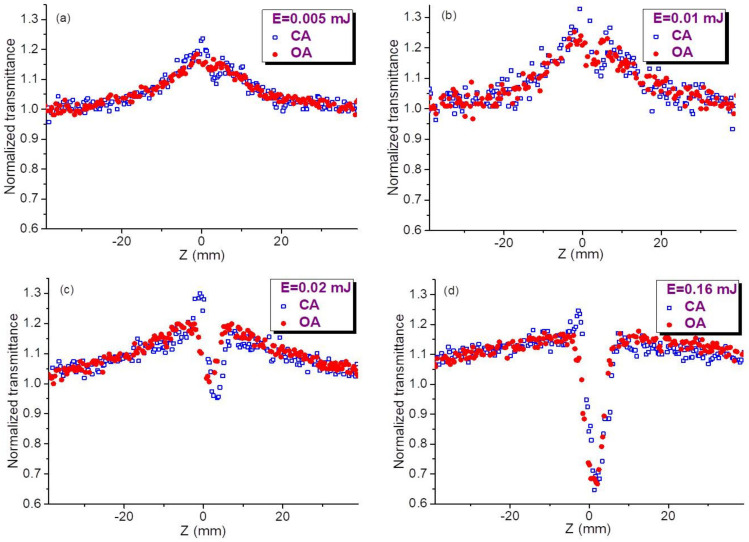
Open-aperture (OA, red filled circles) and closed-aperture (CA, blue empty squares) z-scans of InP@ZnS CQDs using 2.5 times larger concentration of QDs (*C* = 5 × 10^−6^ M) compared with the case shown in Figure 2. These z-scans were measured at different energies of 532 nm pulses. (**a**) 0.005 mJ, (**b**) 0.01 mJ, (**c**) 0.02 mJ, (**d**) 0.16 mJ.

Below we show two groups of z-scans measured using different molar concentrations of QDs in colloidal suspensions (*C* = 2 × 10^−6^ M, Figure 2, and *C* = 5 × 10^−6^ M, Figure 3). We show that the 2.5-fold difference in concentration leads, from one hand, to the growth of SA and, from other hand, to the growth and saturation of the refractive nonlinearities and RSA.

Figure 2 presents four pairs of OA and CA z-scans measured at different energies of 10 ns pulses using smaller concentration of QDs (*C* = 2 × 10^−6^ M). In the case of OA measurements, the saturable absorption manifesting the growth of CQD transmittance in the vicinity of the focal plane in the case of small energies of probe pulses (*E* = 0.005 mJ, Figure 2a, red filled circles) gradually decreased with the growth of the energy of 532 nm radiation (see red filled circles in Figure 2b–d). The saturation of this process of negative nonlinear absorption follows with the appearance of the valley demonstrating the manifestation of the gradual influence of RSA. This modification of OA z-scans at different energies of 532 nm pulses shows the competition of two nonlinear absorptive processes (SA and RSA) when, at the highest used energies (*E* = 0.16 mJ, Figure 2d) the valley attributed to the latter process goes down to *T* = 0.7, while the former process becomes less pronounced.

The nonlinear refraction is barely seen in Figure 2a (*E* = 0.005 mJ, blue empty squares), while gradually demonstrating a difference between the peak and valley in CA curves for larger energies of laser pulses (Figure 2b–d). The self-defocusing properties of CQDs, when peak precedes valley, are clearly seen in all CA curves of Figure 2. Simultaneously with the growth of pulse energy the joint influence of nonlinear refractive and nonlinear absorptive properties of colloidal solution leads to the prevalence of the valley over peak in those CA curves. Like in the case of OA measurements, the growth of joint influence of these processes gradually increases and then saturates. One can compare the approximately two-fold growth of the difference in the transmittances at peak and valley (Δ*T*) of CA curves in the case of using 0.01 and 0.02 mJ pulses. Further growth of pulse energy notably decreases this proportion. Particularly, the 8-fold increase of pulse energy, from 0.02 to 0.16 mJ, causes only the two-fold growth of Δ*T* (compare CA curves in Figure 2c,d).

These energy-dependent measurements of OA and CA z-scans determine the most suitable parameters of probe pulses (0.01–0.02 mJ) at which the saturation of studied nonlinear optical processes caused by the influence of additional effect does not prevent the accurate determination of the nonlinear refraction indices, nonlinear absorption coefficients, and saturated intensities of studied QDs using the theoretical relations of z-scans. The liquid component of colloidal solution (chloroform) did not show the nonlinear optical effects at the used experimental conditions, while being analyzed separately.

Similar pattern of z-scans was observed in the case of 2.5 times larger concentration of QDs in the colloidal suspension (*C* = 5 × 10^−6^ M, Figure 3). The error bars for Figure 2 and Figure 3 were estimated to be 10% for each presented value of normalized transmittance, which represents the averaging of 20 separate measurements. The main difference in these two groups of measurements (Figure 2 and Figure 3) is a growing influence of SA in the latter case. The approximate similarity of OA and CA dependences in Figure 2d and Figure 3d points out the suppression of the nonlinear optical response of studied species at the higher energies of probe pulses.

The optical damage threshold of studied CQDs using 10 ns, 532 nm probe pulses was determined to be 8 × 10^9^ W cm^−2^. This value of optical damage was more than one order of magnitude larger than the intensities used during our measurements of the optical nonlinearities of samples.

Based on above assumption, our further analysis of OA and CA curves using the fitting procedures of experimental data and theoretical approaches was carried out using the moderate energies of probe pulses (0.01–0.02 mJ).

## 4. Optical Limiting and Nonlinear Scattering Studies

Our studies of core-shell suspensions demonstrate the effectiveness of using the long (10 ns) pulses for observation of OL effect. Here we analyze the OL effect in the presence of SA and RSA. Our OA studies revealed that, with the growth of pulse energy, the initially appearing effect of saturable absorption reverts to the manifestation of RSA that efficiently limits the intensity of propagated pulses.

**Figure 4:**
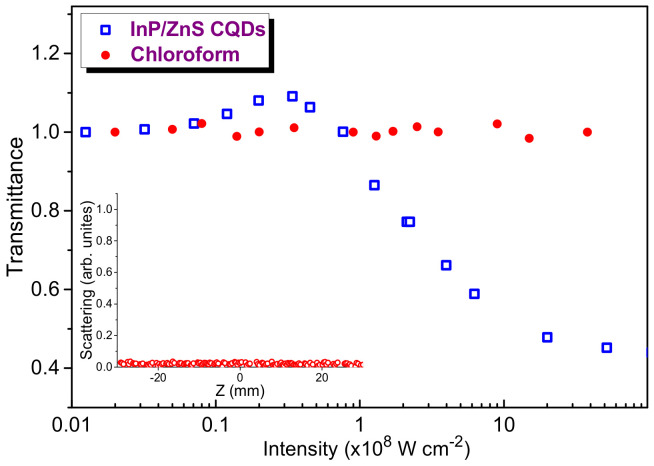
Optical limiting of 532 nm emission at different intensities of probe pulses. Red filled circles: intensity-dependent transmittance of chloroform. Blue empty squares: the same for InP@ZnS CQDs. Inset: dependence of scattered emission on the position of the 1-mm thick cell with regard to the focal plane of the focused probe pulses.

Below we present our studies of OL in InP@ZnS CQDs (*C* = 5 × 10^−6^ M) using 10 ns, 532 nm probe radiation (Figure 4, blue empty squares). Initially, the linear dependence between the input and output pulses was sustained up to the manifestation of SA (7 × 10^6^ W cm^−2^). At relatively small range of intensities (7 × 10^6^–4 × 10^7^ W cm^−2^) the OL curve shown in Figure 4 demonstrates an increase of transmittance with the growth of laser intensity. Then, starting from *I* = 8 × 10^7^ W cm^−2^, a gradual decay of transmittance with the increase of 532 nm probe pulse intensity up to 2 × 10^9^ W cm^−2^ was observed, which then was slowed down until the maximal used intensity during OL experiments (5 × 10^9^ W cm^−2^). This intensity was close to the optical damage of QDs. The limiting of propagated 532 nm radiation can be attributed to the influence of RSA and/or nonlinear scattering. At stronger intensities of probe pulses, some additional nonlinear optical processes can probably play important role. Another reason for the decrease of the OL slope at higher intensities can be the additional process of saturable absorption on the higher-excited states at the intensities *I* > 10^9^ W cm^−2^. The role of main liquid component in OL of CQDs suspension (chloroform) was analyzed at different intensities of the probe pulses (Figure 4, red filled circles). One can see that, along the whole range of used intensities of 532 nm pulses, the normalized transmittance remained unchanged, thus manifesting the absence of nonlinear absorption in the chloroform.

The optical, nonlinear optical and OL properties of small-sized species strongly depend on their size and shape as well as the resonances related with absorption peaks. Since the excitonic peak (~575 nm) lies close to the wavelength of probe pulses, the influence this broadband resonance on the nonlinear refractive indices and nonlinear absorption coefficients at 532 nm tends to be significant.

We did not observe the nonlinear scattering in the case of 532 nm probe pulses. The inset in Figure 4 shows the pattern of unchanged scattering in the focal plane of 532 nm radiation along the whole range of used intensities of probe pulses. Probably, relatively small sizes of QDs (≤4 nm) did not allow the observation of this effect at the used experimental conditions. Thus the nonlinear scattering cannot be considered as the mechanism of the optical limiting at this wavelength and pulse duration.

## 5. Determination of the Nonlinear Optical Parameters of InP@ZnS CQDs

**Figure 5:**
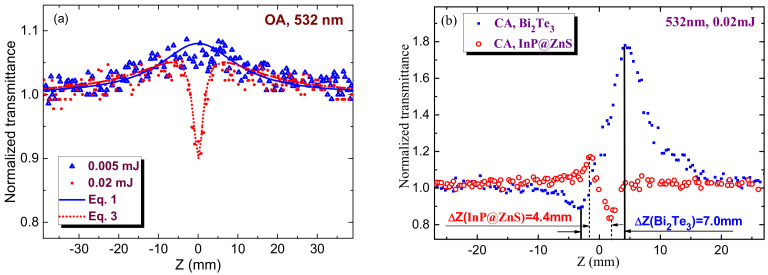
(**a**) Fitting of OA z-scans of core-shell QDs at two energies of probe pulses. Blue solid curve and red dotted curve correspond to the 0.005 and 0.02 mJ pulse energies, respectively. (**b**) CA z-scans of Bi_2_Te_3_ nanoparticles suspension (blue filled squares) and 3.5 nm InP@ZnS core-shell QDs (red empty circles) using 0.02 mJ probe pulses. The corresponding distances between peaks and valleys of these scans were 7 and 4.4 mm.

The fitting procedure to our experimental data included the joint influence of different processes. The probable reason of the noise in data presented in Figure 5a can be some instability of laser intensity from pulse to pulse. This instability does not prevent the fitting without smoothening of experimental data. Below we address this procedure in the case of OA measurements of CQDs (*C* = 5 × 10^−6^ M) at two pulse energies (red filled circles at 0.005 mJ, Figure 3a and 0.02 mJ, Figure 3c). At small energies of probe pulses (Figure 5a, blue empty triangles), the dominating nonlinear optical process is SA, while the role of RSA is almost insignificant.

The following formulas were used to fit our z-scans. The OA z-scan shown in Figure 5a for the case of 0.005 mJ pulses was fitted using the normalized transmittance

*T*(*z*) = [1 + *I*_0_/*I*_sat_ × (1 + *z*^2^/*z*_o_^2^)]^0.5^ (1)

Here *I*_o_ and *I*_sat_ are the laser radiation intensity at the focal plane and saturation intensity and *z*_o_ is the Rayleigh length of the focused radiation, *z*_o_ = *π*(*w*_o_)^2^/*λ*, *w*_o_ is the beam waist radius, and *λ* is the wavelength of probe radiation.

To accurately fit the experimental data of SA one has to chose the conditions when this process appears at unsaturated regime (such as those shown in Figure 2a–c and Figure 3a–c). The SA data in the case of 0.005 mJ pulses (blue empty triangles, Figure 5a) were fitted with Equation (1), which allowed determining *I*_0_/*I*_sat_ = 0.08 and *I*_sat_ to be ~3.7 × 10^8^ W cm^−2^ taking into account the intensity of probe radiation in the focal plane (3 × 10^7^ W cm^−2^).

The increase of pulse energy from 0.005 to 0.02 mJ allowed observation of the valley in OA z-scan (Figure 5a, red filled circles). RSA is a commonly reported mechanism of the nonlinear absorption of QDs. In most cases SA follows by RSA.

The latter process can be fitted by the following relation

*T*(z) = *q*(z)^−1^ ln [1 + *q*(z)] (2)

where *q(z)* = *β I(z) L_eff_*/[1 + (*z_o_^2^*/*z^2^*)], *L_eff_* = [1 − exp (−*α*_o_*L*)]/*α*_o_ is the effective length of the sample, *α*_o_ is the linear absorption coefficient, and *L* is the thickness of the studied sample (*L* = 1 mm in our case).

The joint influence of saturable absorption and reverse saturable absorption is a frequently reported process in molecular, nanoparticle, and quantum dot media. Joint influence of SA and RSA can be considered as the multiplication of the transmittances attributed to those two processes:

*T*(*z*) = [1 + *I*_0_/*I*_sat_ × (1 + *z*^2^/*z*_0_^2^)]^0.5^ × *q*(z)^−1^ × ln [1 + *q*(z)] (3)

The corresponding fitting of Equation (3) and experimental data at *E* = 0.02 mJ (red dotted curve) allows determining the nonlinear absorption coefficient (*β* = 4 × 10^−8^ cm W^−1^) attributed to the influence of RSA.

The definition of nonlinear refractive index of this suspension is a rather difficult procedure taking into account the necessity in determining the mechanism leading to this process. In Figure 5b, we show two CA z-scans, one corresponding to the data shown in Figure 2c for 3.5 nm InP@ZnS core-shell QDs (red empty circles) and another for Bi_2_Te_3_ nanoparticles suspension (blue filled squares). The latter species served for comparison of different mechanisms leading to the nonlinear refraction. The discussion and calculation of the nonlinear refractivity will be presented in Section 6.

## 6. Discussion

In the case of single-atomic species, when quantum confinement does not enhance the nonlinear optical response, the closeness of absorption edge cannot be considered as a reason of incorrectness in determination of the nonlinear optical characteristics of studied sample. Much stronger response from nanoparticles allows the observation of the nonlinear optical processes, which can be enhanced in the vicinity of the excitonic bands (like in our case when 532 nm pump becomes close to the excitonic band of InP cores centered at ~575 nm). In that case one can expect the resonance-related enhancement of third-order susceptibility. TPA unlikely can be considered as a mechanism of positive nonlinear absorption. The rather suitable process in that case is a reverse saturable absorption. The proof for this consideration is the appearance of saturable absorption followed by “saturation” of this process and manifestation of reverse saturable absorption at higher energies of nanosecond pulses.

The application of low pulse repetition rate of probe radiation is a well-known receipt to diminish the role of heat accumulation during propagation of laser pulses through the medium. Our experiments were carried out at very low (1 Hz) pulse repetition rate, which allowed us totally excluding the role of heat accumulation induced thermal lens in determination of the value of *γ*. Our experiments were carried out up to 20 Hz to decrease the collection time of experimental data, and we did not observe the variation of this parameter, which should be notably increased, provided the heat accumulation plays important role at current experimental conditions. Because of this below we are considering another (acoustic) mechanism of heat-related self-defocusing and compare it with the heat accumulation mechanism.

The appearance of thermal lens can be attributed to the two processes resulting in heat-induced negative nonlinear refraction. Those are (a) the thermal accumulation effect caused by heat accumulation in the focal area and (b) acoustic density variation. The first process can be caused by either nonlinear or linear absorption. The time scale of this process is of the order of 1 µs, which is notably less that the period between two pulses in our experiments (1 s). As mentioned, the thermal accumulation did not play important role in present experiments, which was proved by our variations of pulse repetition rate that did not show any significant modifications in the shape of CA z-scans. Second process is related with the density variation along the period of propagation of the acoustic wave from the focal point to the boundaries of the focal volume the timescale of which determines by the relation *t* = *w*_o_/*ʋ*, where *w*_o_ is the beam waist radius and *ʋ* is the velocity of sound in the colloidal suspension. Commonly, this period is of the order of 1 to 10 ns, which points out that, during propagation of used pulse (10 ns), the acoustic wave can decrease the density of medium. In that case the trailing part of pulse can underwent the influence of the acoustic wave induced variation of density on the refractive properties of the medium.

This effect has been demonstrates earlier in a few media (liquids and nanoparticle suspensions, see for example [24]) and can be manifested, apart from the specific shape of CA z-scan, by the variation of the temporal shape of the laser pulse propagated through the studied medium. The trailing part of 10-ns pulse will be suppressed while measuring it after propagation of the aperture placed in front of the time-resolved registrar. Thus, building of the thermal lens in our case can be dominated by acoustic density changes rather than thermal accumulation effects. Correspondingly, the influence of the thermal effect attributed to the acoustic wave propagation should not be neglected in similar experiments carrying out using nanosecond lasers.

The critical parameter of the experiments using nanosecond pulses is the absorption, either linear or nonlinear, of the medium. Our estimates show that the self-defocusing can be manifested 5 ns from the beginning of laser pulse propagation through the studied medium.

Another confirmation of the thermal nature of observed self-defocusing is the distance between the peak and valley in the CA z-scan. In the case of Kerr-related nonlinearity, this distance (ΔZ) has the following relation with the Rayleigh length of the focused radiation (z_o_): ΔZ ≈ 1.7z_o_ [23]). Such a relation was maintained in the case when Kerr-related nonlinearity showed a prevailing influence over the thermal-induced refractive nonlinearity (Figure 5b, blue filled squares showing our separate CA z-scan experiments using 532 nm, 10 ns pulses and bismuth telluride nanoparticles suspension at similar focusing conditions). In those experiments with Bi_2_Te_3_ nanoparticles suspension we observed the positive nonlinear refraction and saturable absorption of 532 nm, 10 ns pulses. ΔZ in that case was equal to 7 mm, which almost obeyed the above relation between the Rayleigh length and valley-to-peak distance along the Z axis for the Kerr-induced refractive nonlinearities (ΔZ ≈ 1.75z_o_ in our case, z_o_ during our experiments was equal to 4 mm). This study showed that the fast (electronic) nonlinearity leading to positive nonlinear refractivity was notably larger than the slow (thermal) nonlinearity inducing the self-defocusing of propagated nanosecond pulse.

Another pattern was observed in the case of InP@ZnS core-shell quantum dots (Figure 5b, red empty circles). The peak-to-valley separation along Z axis in that case was 4.4 mm, which corresponds to the relation ΔZ ≈ 1.1z_o_. In accordance with studies [25], such a relation is attributed to the thermo-optical nonlinearity. Thus we can attribute the observed process of self-defocusing in InP@ZnS core-shell quantum dots to the thermal effect. Notice the absence of thermal lens building in the case of the pure chloroform representing the main liquid component of the studied suspension. Meantime, the role of Kerr effect in the case of InP@ZnS core-shell quantum dots is hardly can be separated from the thermal process, though in the case of molecular structures the role of other Kerr-related effects, such as molecular orientational process, can be notably increased in the case of nanosecond pulses with regard to the pure electronic Kerr effect. We assume that our quantum dots have a symmetric shape and cannot be treated as the anisotropic structures allowing the reorientation during laser pulse and thus do not further consider this process.

In most cases, when the probe radiation lies in the visible range, the quantum dots by themselves demonstrate the negative Kerr-related nonlinear refractive index while measuring by short (ps and fs) pulses. The consideration of fast processes in semiconductors is presented in [26] using Kramers-Kronig relations. Authors of [26] found that the nonlinear refraction changes sign at *ħω/E*_g_ = 0.69 (*ħ* is the Planck’s constant, *ω* is the frequency of laser radiation, and *E*_g_ is the band gap energy of semiconductor). Particularly, once this parameter becomes larger than 0.69 the medium starts showing the self-defocusing properties. In this connection, the sign of nonlinear refraction can be changed once one considers bulk material and nanoparticles of the same elemental consistence. Correspondingly these species demonstrate the negative sign of *γ*. However, smaller nanocrystallites may demonstrate a large blue shift in the absorption edge that leads to the variations in effective band gap. Thus the parameter *ħω/E*_g_ becomes less than 0.69 and the smaller nanoparticles can demonstrate the self-focusing since their band gap became notably increased. This reversion of optical nonlinearities can be understood in terms of quantum size effect resulting from the confinement of an electron-hole pair in a small volume.

Similarly, the Kramers-Kronig model provides some clues in definition of the sign transformation of *γ* from self-focusing to self-defocusing and vice versa once one uses different wavelengths of probe pulses [26]. The crucial parameter in this model is the band gap of semiconductor. Once the radius of semiconductor nanoparticles decreases their band gap can be increased. It is not clear whether it is correct treating InP@ZnS core-shell quantum dots as the matter possessing the semiconductor properties. However, some assumptions can be retrieved once one considers the similarity of the properties of different morphological structures containing similar elemental components. The band gap of QDs in our case was estimated to be 1.89 eV. The corresponding *ħω/E*_g_ value for InP@ZnS core-shell quantum dots at the used wavelength of probe pulses was calculated to be notably larger than 0.69 (*ħω/E*_g_ = 1.23). Thus, one can expect a negative sign of *γ* in these QD containing suspensions, once one considers only fast (Kerr-related) processes in the case of 532 nm probe pulses.

Thus both thermal-related and Kerr-related considerations point out that the studied species should possess the self-defocusing properties. The question arises which one among two (Kerr- and heat-related) processes prevails in our studies. Previous studies show that the species with *ħω/E*_g_ close to 0.69 demonstrate strong Kerr-related nonlinear refraction and vice verse. In our case (*ħω/E*_g_ = 1.23) one can hardly expect the large Kerr-related *γ* of studied species. Earlier, the conclusion on the prevailing role of heat-related process in InP@ZnS core-shell quantum dots was reported in [27] in the case of 4 ns, 532 nm probe pulses. Notice their finding of the positive sign of *γ* at the same wavelength of InP@ZnS core-shell quantum dots dissolved in toluene in the case of femtosecond probe pulses was attributed to the prevailing influence of the toluene, which modifies the negative sign of nonlinear refraction of InP@ZnS core-shell quantum dots towards the positive sign in the case of InP@ZnS+toluene suspension.

Authors of [27] found the lifetime (10.7 ns) using transient absorption measurements, which was attributed to the recombination time of electrons in the conduction band and the holes in the valence band. Therefore, the excited-state absorption, which can play a crucial role in both nonlinear absorption and heat-related nonlinear refraction, cannot be ignored during nanosecond pulses excitation. Authors of [27] also concluded that the switch mechanism of nonlinear absorption with the excitation of nanosecond pulses for InP/ZnS QDs was attributed to the excited-state absorption. Similar process was observed in our case (see Figure 5a). The analysis of their CA z-scans allows defining that the self-focusing in the case of femtosecond pulses attributed to the Kerr effect in toluene (ΔZ ≈ 9 mm) transforms to the heat-related self-defocusing (ΔZ ≈ 5.5 mm) in the case of nanosecond pulses, very similar to our observation and conclusion arising from the comparison of two above shown CA z-scans of Bi_2_Te_3_ nanoparticles and InP@ZnS QDs. Since in both cases the duration of used nanosecond pulses was close to the recombination time of electron and hole, one can attribute the self-defocusing to the thermal effect. Our calculations of *γ* (−2 × 10^−12^ cm^2^ W^−1^; see below) showed that this parameter was one order of magnitude smaller than the measurements of InP@ZnS QDs presented in [27] (−1.6 × 10^−11^ cm^2^ W^−1^), which can be attributed to rather larger molar concentration of the used colloidal suspension in the latter case (0.1 mg/mL in the toluene solvent).

The application of relatively strong pulses (0.16 mJ) led to saturation of all nonlinear optical processes. The energy-dependent experiments were carried out to demonstrate the range of pulse energies at which no saturation was observed. We defined the 0.01–0.02 mJ range at which the nonlinear processes change almost linearly with the growth of laser energy. Correspondingly, the fitting and calculations of nonlinear parameters were performed using low energies of pulses (0.005 and 0.02 mJ; see Figure 5a).

As it was mentioned, the peak and valley in CA z-scan (Figure 5b, red empty circles) becomes closer compared with the conventional cubic process, with a separation ΔZ ≈ 1.1z_o_. Notice a difference between this separation and the one obtained for the cubic nonlinearity (ΔZ ≈ 1.7z_o_). The “purely” thermal effects related with linear absorption normally show a separation larger than the 1.1z_o_ value [28]. Thus one can consider the higher-order process resulting from a nonlinear absorption giving rise to the thermooptical nonlinearity. The question is: what could be a possible mechanism of positive nonlinear absorption in our case? Present measurements show that the only process, which can cause the nonlinear absorption, is RSA. The positive nonlinear absorption starts appearing on this graph (Figure 5b, red empty circles) at 0.02 mJ pulse energy. Another question is: how to fit this experimental result? And is it necessary to fit our data with some thermo-optics-related theoretical curve to determine *γ* or there is a way to determine this parameter by simpler method?

The standard equations commonly used to fit the CA data were obtained based on a cubic nonlinearity (i.e., *χ*^(3)^ effect) [29]. Meanwhile, a simplified equation (ΔT_pv_ ≈ 0.4(1 − S)^0.25^|ΔΦ_o_|) is commonly used for the direct calculation of the nonlinear refractive index at the conditions when the role of nonlinear absorptive effects becomes insignificant [26]. Here ΔT_pv_ is the normalized difference between peak and valley transmissions, S is the transmission of the aperture in the case of CA scheme, ΔΦ_o_ = *kγI*_0_*L*_eff_ and *k* = 2*π*/*λ*. A similar analysis was performed for higher-order nonlinearities [23]. Authors of [23] treated the nonlinearities encountered in semiconductors where the index of refraction is altered through charge carriers generated by TPA or RSA as a sequential *χ*^(3)^: *χ*^(1)^ effect, which can be considered as a fifth-order nonlinearity [30]. Authors of [23] found that, for a fifth-order effect, the peak and valley should be separated by 1.2z_o_ as compared to 1.7z_o_ obtained for the third-order effect. The process observed in our case (i.e., nonlinear refraction as a consequence of the thermal lens formation induced by RSA) can also be treated as a sequential effect. Thus our CA z-scan, approximately obeying the above-mentioned peak-to-valley separation rule (ΔZ ≈ 1.2z_o_) for such sequential effect, can be attributed to the fifth-order process.

The relation for the peak-to-valley transmittance for a small aperture (S = 0) in that case is as follows: ΔT_pv_ ≈ 0.21|ΔΦ_o_|. Here ΔΦ_o_ = *kηI*_o_^2^*L*_eff_ and *η* is the fifth-order nonlinear refractive index, *η* = *γ*/*I*_o_. The third-order nonlinear refractive index calculated using this formula and data presented in Figure 5b for InP@ZnS QDs was equal to *γ* = −2 × 10^−12^ cm^2^ W^−1^. The corresponding figure of merit for the studied colloidal suspension of quantum dots is |*γ*/*C*| = 1 × 10^−6^ cm^2^ W^−1^M^−1^.

Below we address some peculiarities of the transient absorption dynamics and energy diagram of InP@ZnS QDs earlier analyzed in [27]. The lifetime of 4.7 ps was attributed to the relaxation of hot exciton, while the lifetimes of 82 ps and 10.7 ns were attributed to the excited-state lifetime of the electron and the recombination time of electrons in the conduction band and the holes in the valence band respectively. In [31], the ultrafast transient absorption spectroscopy provided the understanding of the carriers’ dynamics occurring within picosecond time scale. The excited states lifetimes in InP@ZnS QDs were measured to be in the range of 170 to 230 ps, which are differ from the measurements presented in [27].

The energy diagram demonstrating schematic illustration of carrier injection and transport is discussed in [31]. Photoexcited carrier first injected to ZnS shell in 2 ps and then relaxed to alloyed layer between ZnS shell and InP core in 7.4 ps, with further relaxation to different energy levels in InP core in about 180 ps. After optical excitation, electron and hole will undergo a fast thermalization process before the formation of an exciton, then relax to a K = 0 state before radiation. After relaxation or cooling, the charged excitons and neutral excitons recombine in 4.1 ns and 26.7 ns, respectively.

TPA at these conditions can hardly be considered as a preferable mechanism of nonlinear absorption in the case of 532 nm, 10 ns pump. The most probable mechanism of absorption, as we pointed out, is RSA. TPA in InP/ZnS QDs can be realized in the wavelength range between 750 nm and 975 nm, while in the shorter wavelength range the TPA cross section was notably decreased [13].

In our case, the near resonant third-order nonlinearities of InP@ZnS QDs were measured at 532 nm within the absorption band. The growth of transmittance at small energies of probe 532 nm pulses indicates that SA occurred in InP@ZnS QDs, similarly to the earlier studies of these species using 100 fs pulses [14]. Meanwhile, the studies using nanosecond pulses showed that the available ground state carriers deplete even at relatively small intensities of excitation [27].

Apparently, the relative position of the core and shell levels provides the SA. From the fitting of the normalized transmittance (solid curve in Figure 5a), *β*_SA_ attributed to SA was determined to be −1.4 × 10^−9^ cm W^−1^, which is larger than the same parameter measured using the 527 nm, 100 fs pulses (−5.9 × 10^−10^ cm W^−1^, [14]). Additionally, the heat-induced negative nonlinear refractive index of InP@ZnS CQDs retrieved during our studies (−2 × 10^−12^ cm^2^ W^−1^) was a few orders of magnitude larger than the Kerr-induced index determined at 800 nm in the case of 100 fs pulses (−7.1 × 10^−16^ cm^2^ W^−1^). Thus one can assume the importance of the temporal and spectral characteristics of the probe radiation in determination of the nonlinear optical characteristics of studied species. Particularly, the difference in the nonlinear refractive properties of core-shell structure can be attributed to the closeness of the probe pulse’s wavelength and excitonic band in the case of 532 nm pulses compared with the 800 nm probe pulses. Another reason in the difference of the nonlinear refractive response of InP@ZnS CQDs in the reported and our cases can be attributed to different experimental conditions (sizes of QDs, molar concentration, specific defects of core-shell structures, etc.) as well as the involvement of additional mechanisms, other than the pure electronic Kerr effect responsible for the nonlinear refraction under the action of ultrashort laser pulses [32,33].

The nonlinear optical parameters of studied CQDs using 532 nm, 10 ns probe pulses are summarized in Table 1.

The observed effect of OL is probably related to the charge transfer. The relative position of the energy levels of the two semiconductors is still poorly understood in spite of their extensive studies [31]. The approximate location of some of those energy levels is given in [34]. One can assume that ZnS has the ground state energy 7.0 eV. In this case, taking into account the size effect, the transfer of the electron from InP to the conduction states of ZnS is likely. It is also possible to transfer the charge to specific defect levels that are suitable in their energy and charge for this purpose.

The strong nonlinear optical response of studied core-shell structures allows expecting them to be efficient emitters of high-order harmonics during propagation of ultrashort pulses through the core-shell QDs-containing plasmas, similarly to recently demonstrated advantages of metal sulfide QDs as the suitable media for harmonics generation in the extreme ultraviolet range.

## 7. Conclusions

The novelty of this study is related with broader consideration of the thermal-related nonlinear refractive processes, analysis of the influence of particles concentration and pulse energy on the nonlinear optical response, distinction of the role of acoustic and accumulative processes in formation of thermal lens, analysis of optical limiting and nonlinear scattering in studied quantum dots, and determination of various nonlinear optical parameters of InP@ZnS core-shell colloidal quantum dots.

We have reported the studies of the low-order optical nonlinearities of InP@ZnS core-shell colloidal quantum dots. The nonlinear refraction and nonlinear absorption coefficients (*γ* = −2 × 10^−12^ cm^2^ W^−1^, *β* = 4 × 10^−8^ cm W^−1^) of these CQDs were determined using 10 ns, 532 nm pulses. The saturable absorption (*β*_SA_ = −1.4 × 10^−9^ cm W^−1^, *I*_sat_ = 3.7 × 10^8^ W cm^−2^) in the 3.5-nm-sized CQDs dominated at small intensities of the probe pulses (*I* ≤ 7 × 10^7^ W cm^−2^) followed by reverse saturable absorption at higher laser intensities. We have reported the optical limiting studies using these CQDs showing the suppression of propagated nanosecond radiation in the intensity range of 8 × 10^7^–2 × 10^9^ W cm^−2^. The role of nonlinear scattering is considered using off-axis z-scan scheme, which demonstrated the insignificant role of this process along the whole range of used intensities of 532 nm pulses. We have discussed the thermal related processes of nonlinear refraction in studied CQD samples.

## Figures and Tables

**Table 1:** Summary of the nonlinear optical measurements of InP@ZnS CQDs. *λ* is the wavelength of probe pulse (532 nm), *ħ* is the Planck’s constant, *ω* is the frequency of laser radiation, *E*_g_ is the energy band gap of studied QDs, *γ* is the nonlinear refractive index, C is the molar concentration, *β*_SA_ is the nonlinear absorption coefficient attributed to saturable absorption, *β* is the nonlinear absorption coefficient attributed to reverse saturable absorption, *I*_sat_ is the saturated intensity, and Re*χ*^(3)^ is the real part of third-order nonlinear susceptibility.

*λ*	Size of QDs	*ħω*/*E*_g_	*γ*	|*γ*/*C*|	*β*_SA_ (SA)	*β* (RSA)	*I*_sat_	Re*χ*^(3)^
532 nm	3.5 nm	1.23	−2 × 10^−12^ cm^2^ W^−1^	1 × 10^−6^ cm^2^ W^−1^ M^−1^	−1.4 × 10^−9^ cm W^−1^	4 × 10^−8^ cm W^−1^	3.7 × 10^8^ W cm^−2^	3 × 10^−13^ esu

## Data Availability

Data underlying the results presented in this paper are not publicly available at this time but may be obtained from the authors upon reasonable request.
